# Supplementary material for: Predicting student and instructor e-readiness and promoting e-learning success in online EFL class during the COVID-19 pandemic: A case from China
Source: PLoS One. 2023 May 8;18(5):e0284334. doi: 10.1371/journal.pone.0284334 (PMC10166505; doi:10.1371/journal.pone.0284334)
Supplement: S1 File — (ZIP) [file pone.0284334.s001.zip › Supporting information/questionnaire report σ£¿τ║┐Φï▒Φ»¡σ¡aΣ╣áτ¢╕σà│Θù«σì╖ΓÇöΓÇöC.Y. Yang∩╝ìΘ╗ÿΦ«ñμèÑσæè.doc]

**在线英语学习相关问卷——C.Y. Yang**

1.您的身份（	）	[单选题]

| 选项 | 小计 | 比例 |
| --- | --- | --- |
| A.学生 | 6024 | 77.47% |
| B.教师 | 1752 | 22.53% |
| 本题有效填写人次 | 7776 |  |

2.您的性别:（	）	[单选题]

| 选项 | 小计 | 比例 |
| --- | --- | --- |
| A.男 | 3325 | 55.2% |
| B.女 | 2699 | 44.8% |
| 本题有效填写人次 | 6024 |  |

3.您的年级:（	）	[单选题]

| 选项 | 小计 | 比例 |
| --- | --- | --- |
| A.大学一年级 | 1453 | 24.12% |
| B.大学二年级 | 1691 | 28.07% |
| C.大学三年级 | 1567 | 26.01% |
| D.大学四年级 | 1313 | 21.8% |
| 本题有效填写人次 | 6024 |  |

4.您现在还有英语课吗? [单选题]

| 选项 | 小计 | 比例 |
| --- | --- | --- |
| A.有 | 5914 | 98.17% |
| B.无 | 110 | 1.83% |
| 本题有效填写人次 | 6024 |  |

5.您是在城市地区/农村地区学习在线英语课程？	[单选题]

| 选项 | 小计 | 比例 |
| --- | --- | --- |
| A.城市 | 5060 | 85.56% |
| B.农村 | 854 | 14.44% |
| 本题有效填写人次 | 5914 |  |

6.您所就读的大学	。	[填空题]

填空题数据请通过下载详细数据获取

7.您就读的大学所在的省份	。	[填空题]

填空题数据请通过下载详细数据获取

8.您所就读的大学属于	。	[单选题]

| 选项 | 小计 | 比例 |
| --- | --- | --- |
| A. 部属本科院校（Directly under MOE） | 1407 | 23.79% |
| B.省部共建/省属本科院校 (Co-construction by provinces and ministries/Under provincial department of education) | 3099 | 52.4% |
| C.市属本科院校(Municipal college) | 437 | 7.39% |
| D.民办本科院校(Private undergraduate colleges) | 684 | 11.57% |
| E.公办职业院校（State-owned vocational colleges ） | 287 | 4.85% |
| 本题有效填写人次 | 5914 |  |

9.您是否有前期在线学习体验/经验?持续时间多长? [单选题]

| 选项 | 小计 | 比例 |
| --- | --- | --- |
| A.有，1-6个月 | 1109 | 18.75% |
| B.有，7-12个月 | 1315 | 22.24% |
| C.有，13-24个月 | 903 | 15.27% |
| D.有，25个月及以上 | 394 | 6.66% |
| E.无 | 2193 | 37.08% |
| 本题有效填写人次 | 5914 |  |

10.您目前的专业是 (中文作答）。	[填空题]

填空题数据请通过下载详细数据获取

11.您常使用何种设备开展网络在线英语课程（	）? [单选题]

| 选项 | 小计 | 比例 |
| --- | --- | --- |
| A.手机（数据流量） | 1304 | 22.05% |
| B.手机（WI-FI） | 1602 | 27.09% |
| C.电脑（WI-FI/宽带） | 1224 | 20.7% |
| D.平板电脑/ipad（WI-FI） | 1697 | 28.69% |
| E.其他 | 87 | 1.47% |
| 本题有效填写人次 | 5914 |  |

12.您是否有考验意向/出国意向（	）	[单选题]

| 选项 | 小计 | 比例 |
| --- | --- | --- |
| A.有 | 1355 | 22.91% |
| B.无 | 4559 | 77.09% |
| 本题有效填写人次 | 5914 |  |

13.您最常用的在线英语学习平台（	）	[单选题]

| 选项 | 小计 | 比例 |
| --- | --- | --- |
| A.腾讯会议 | 1243 | 21.02% |
| B.QQ课堂/QQ直播 | 1193 | 20.17% |
| C.钉钉 | 1393 | 23.55% |
| D.超星学习通 | 668 | 11.3% |
| E.雨课堂 | 846 | 14.31% |
| F.职教云 | 459 | 7.76% |
| G.其他 | 112 | 1.89% |
| 本题有效填写人次 | 5914 |  |

14.我认为我所使用的设备操作简单。（I recognize that the device using for the online EFL class is easy to operate.）	[单选题]

| 选项 | 小计 | 比例 |
| --- | --- | --- |
| A.非常同意 | 1915 | 32.38% |
| B.同意 | 2231 | 37.72% |
| C.不一定 | 1005 | 16.99% |
| D.不同意 | 566 | 9.57% |
| E.非常不同意 | 197 | 3.33% |
| 本题有效填写人次 | 5914 |  |

15. 我认为我所使用的设备对用户很友好，让我有很好的体验感。（I recognize that the device using for the online EFL class is user-friendly.）	[单选题]

| 选项 | 小计 | 比例 |
| --- | --- | --- |
| A.非常同意 | 1998 | 33.78% |
| B.同意 | 1920 | 32.47% |
| C.不一定 | 1026 | 17.35% |
| D.不同意 | 616 | 10.42% |
| E.非常不同意 | 354 | 5.99% |
| 本题有效填写人次 | 5914 |  |

16. 我认为我所使用的设备很稳定、安全。（I recognize that the device using for the online EFL class is stable and secure.）	[单选题]

| 选项 | 小计 | 比例 |
| --- | --- | --- |
| A.非常同意 | 1815 | 30.69% |
| B.同意 | 2001 | 33.83% |
| C.不一定 | 1132 | 19.14% |
| D.不同意 | 597 | 10.09% |
| E.非常不同意 | 369 | 6.24% |
| 本题有效填写人次 | 5914 |  |

17. 我认为我所使用的设备运行很快，不卡顿。（I recognize that the device using for the online EFL class runs smoothly and fast.）	[单选题]

| 选项 | 小计 | 比例 |
| --- | --- | --- |
| A.非常同意 | 1734 | 29.32% |
| B.同意 | 2034 | 34.39% |
| C.不一定 | 1155 | 19.53% |
| D.不同意 | 644 | 10.89% |
| E.非常不同意 | 347 | 5.87% |
| 本题有效填写人次 | 5914 |  |

18.我认为使用网络开展在线英语课程的频率很高（指每周4节课，每节课45分钟）。（I recognize that we have high frequency of online EFL class,generally having 4 classes per week, each class lasting for 45 minutes.) [单选题]

| 选项 | 小计 | 比例 |
| --- | --- | --- |
| A.非常同意 | 1631 | 27.58% |
| B.同意 | 1990 | 33.65% |
| C.不一定 | 1277 | 21.59% |
| D.不同意 | 653 | 11.04% |
| E.非常不同意 | 363 | 6.14% |
| 本题有效填写人次 | 5914 |  |

19.我能独立使用网络开展在线英语课程。（I can have my online EFL class independently. [单选题]

| 选项 | 小计 | 比例 |
| --- | --- | --- |
| A.非常同意 | 1306 | 22.08% |
| B.同意 | 1435 | 24.26% |
| C.不一定 | 1220 | 20.63% |
| D.不同意 | 1138 | 19.24% |
| E.非常不同意 | 815 | 13.78% |
| 本题有效填写人次 | 5914 |  |

20.我对在线英语课堂很满意。（I am satisfied with the online EFL class.) [单选题]

| 选项 | 小计 | 比例 |
| --- | --- | --- |
| A.非常同意 | 1748 | 29.56% |
| B.同意 | 1993 | 33.7% |
| C.不一定 | 1195 | 20.21% |
| D.不同意 | 669 | 11.31% |
| E.非常不同意 | 309 | 5.22% |
| 本题有效填写人次 | 5914 |  |

21.我认为在线英语课堂整体而言是成功的。（The online EFL class has again an overall success. )	[单选题]

| 选项 | 小计 | 比例 |
| --- | --- | --- |
| A.非常同意 | 1701 | 28.76% |
| B.同意 | 1976 | 33.41% |
| C.不一定 | 1218 | 20.6% |
| D.不同意 | 682 | 11.53% |
| E.非常不同意 | 337 | 5.7% |
| 本题有效填写人次 | 5914 |  |

22.我认为在线英语课堂为提高了我的英语学习兴趣。（I think online EFL class arouses my interests in learning English.) [单选题]

| 选项 | 小计 | 比例 |
| --- | --- | --- |
| A.非常同意 | 1564 | 26.45% |
| B.同意 | 2020 | 34.16% |
| C.不一定 | 1271 | 21.49% |
| D.不同意 | 707 | 11.95% |
| E.非常不同意 | 352 | 5.95% |
| 本题有效填写人次 | 5914 |  |

23.我认为在线英语课堂将我和老师们、同学们紧密联系起来了。（I think the online EFL class connects me with my instructors and classmates.) [单选题]

| 选项 | 小计 | 比例 |
| --- | --- | --- |
| A.非常同意 | 1605 | 27.14% |
| B.同意 | 1947 | 32.92% |
| C.不一定 | 1271 | 21.49% |
| D.不同意 | 718 | 12.14% |
| E.非常不同意 | 373 | 6.31% |
| 本题有效填写人次 | 5914 |  |

24.我关注在线英语教学质量与个人学习效果。（I concern for the teaching and learning quality of the online EFL class.) [单选题]

| 选项 | 小计 | 比例 |
| --- | --- | --- |
| A.非常同意 | 1514 | 25.6% |
| B.同意 | 1999 | 33.8% |
| C.不一定 | 1212 | 20.49% |
| D.不同意 | 758 | 12.82% |
| E.非常不同意 | 431 | 7.29% |
| 本题有效填写人次 | 5914 |  |

25.我认为在线英语课程提升了我的英语学业成绩。（I think that online EFL class has improved my English academic success. )	[单选题]

| 选项 | 小计 | 比例 |
| --- | --- | --- |
| A.非常同意 | 1245 | 21.05% |
| B.同意 | 1462 | 24.72% |
| C.不一定 | 1135 | 19.19% |
| D.不同意 | 1108 | 18.74% |
| E.非常不同意 | 964 | 16.3% |
| 本题有效填写人次 | 5914 |  |

26.对于在线英语课程结束后遇到的问题，我可以独立处理。（As for the problems encountered after the EFL class, I can deal with them without any prompt help-desk service.) [单选题]

| 选项 | 小计 | 比例 |
| --- | --- | --- |
| A.非常同意 | 1663 | 28.12% |
| B.同意 | 1975 | 33.4% |
| C.不一定 | 1225 | 20.71% |
| D.不同意 | 707 | 11.95% |
| E.非常不同意 | 344 | 5.82% |
| 本题有效填写人次 | 5914 |  |

27.我认为在线英语课堂提升了我的英语学习能力与水平。（I think that the online EFL class improved my English learning capabilities.) [单选题]

| 选项 | 小计 | 比例 |
| --- | --- | --- |
| A.非常同意 | 1498 | 25.33% |
| B.同意 | 1944 | 32.87% |
| C.不一定 | 1232 | 20.83% |
| D.不同意 | 778 | 13.16% |
| E.非常不同意 | 462 | 7.81% |
| 本题有效填写人次 | 5914 |  |

28.您认为如何可以让您更好的融入在线英语课堂？（How will you be engaged in the online EFL class?) [填空题]

填空题数据请通过下载详细数据获取

29.请选出6个您觉得对您在线英语学习最有价值的学习因素（多选题）:(Please choose 6 of the following items as the most valuable online EFL learning elements.) [多选题]

| 选项 | 小计 | 比例 |
| --- | --- | --- |
| A. Understanding the course materials | 3065 | 51.83% |
| B. Fluent and free internet connect | 2934 | 49.61% |
| C. Beingself-discipline and properly managing time | 3137 | 53.04% |
| D. A silent place for studying | 3118 | 52.72% |
| E. Teacher presence | 3145 | 53.18% |
| F. Understanding the professor | 2455 | 41.51% |
| G. Participating in student-student communications | 3285 | 55.55% |
| H. Teacher-student interaction | 3519 | 59.5% |
| I. Reviewing course content | 3282 | 55.5% |
| J. Completing assignments | 2477 | 41.88% |
| K. Obtaining feedback from professor | 3119 | 52.74% |
| L. Obtaining feedback from assessed work | 2388 | 40.38% |
| M. Taking practice examinations | 2409 | 40.73% |
| N. Practicing problem solving ability | 3230 | 54.62% |
| O. Others | 1006 | 17.01% |
| 本题有效填写人次 | 5914 |  |

30.请您列举您当前面临的在线英语学习的困难（请尽量详细、具体描述）。（Please list some impediments of online EFL learning.) [填空题]

填空题数据请通过下载详细数据获取

31.请您列举提升在线英语学习的建议(角度:硬件设备与技术、人力资源、教学质量、教学内容、教学相关服务、网络、课程设计、教师教学技巧等）:（Please list some recommendations on promoting online EFL class，such as infrastructure and technology, human resources, quality, content and services, network enablers, curriculum design, teacher skills etc.) [填空题]

填空题数据请通过下载详细数据获取

32.请补充您认为该研究需要拓展的角度:（Please list some recommendations on this research in case of not covering those paramount perspectives.) [填空题]

填空题数据请通过下载详细数据获取

33.您的性别:（	）	[单选题]

| 选项 | 小计 | 比例 |
| --- | --- | --- |
| A.男 | 590 | 33.68% |
| B.女 | 1162 | 66.32% |
| 本题有效填写人次 | 1752 |  |

34.您的年龄阶段（ ）	[单选题]

| 选项 | 小计 | 比例 |
| --- | --- | --- |
| A.24—30岁 | 181 | 10.33% |
| B.31—35岁 | 348 | 19.86% |
| C.36—40岁 | 641 | 36.59% |
| D.41—45岁 | 445 | 25.4% |
| E.46岁及以上 | 137 | 7.82% |
| 本题有效填写人次 | 1752 |  |

35.您所工作的大学属于	。	[单选题]

| 选项 | 小计 | 比例 |
| --- | --- | --- |
| A.部属本科院校（Directly under MOE） | 261 | 14.9% |
| B.省部共建/省属本科院校(Co-construction by provinces and ministries/Under provincial department of education) | 824 | 47.03% |
| C.市属本科院校(municipal college) | 185 | 10.56% |
| D.民办本科院校(Private undergraduate colleges) | 365 | 20.83% |
| E.公办职业院校（State-owned vocational colleges ） | 117 | 6.68% |
| 本题有效填写人次 | 1752 |  |

36.您是否有在线授课经验?您开展在线英语教学年限:（ ）	[单选题]

| 选项 | 小计 | 比例 |
| --- | --- | --- |
| A.有，1-6个月 | 227 | 12.96% |
| B.有，7-12个月 | 482 | 27.51% |
| C.有，13-24个月 | 679 | 38.76% |
| D.有，25个月及以上 | 362 | 20.66% |
| E.无 | 2 | 0.11% |
| 本题有效填写人次 | 1752 |  |

37.您开展的是同步/直播的在线英语课堂、异步英语课堂，还是两者结合?（Is the online EFL class Synchronous, Asynchronous, or Combination of the two?) [单选题]

| 选项 | 小计 | 比例 |
| --- | --- | --- |
| A.同步的（Synchronous） | 741 | 42.29% |
| B.异步的（Asynchronous） | 222 | 12.67% |
| C.同步与异步相结合（Combination of the two） | 789 | 45.03% |
| 本题有效填写人次 | 1752 |  |

38.您最常用的在线英语授课平台（	）	[单选题]

| 选项 | 小计 | 比例 |
| --- | --- | --- |
| A.腾讯会议 | 389 | 22.2% |
| B.QQ课堂/QQ直播 | 422 | 24.09% |
| C.钉钉 | 463 | 26.43% |
| D.超星学习通 | 102 | 5.82% |
| E.雨课堂 | 230 | 13.13% |
| F.职教云 | 128 | 7.31% |
| G.其他 | 18 | 1.03% |
| 本题有效填写人次 | 1752 |  |

39.我所使用的设备操作简单。（I recognize that the device using for the online EFL class is easy to operate.）	[单选题]

| 选项 | 小计 | 比例 |
| --- | --- | --- |
| A.非常同意 | 597 | 34.08% |
| B.同意 | 651 | 37.16% |
| C.不一定 | 291 | 16.61% |
| D.不同意 | 156 | 8.9% |
| E.非常不同意 | 57 | 3.25% |
| 本题有效填写人次 | 1752 |  |

40. 我认为我所使用的设备对用户很友好，让我有很好的体验感。（I recognize that the device using for the online EFL class is user-friendly.）	[单选题]

| 选项 | 小计 | 比例 |
| --- | --- | --- |
| A.非常同意 | 508 | 29% |
| B.同意 | 584 | 33.33% |
| C.不一定 | 375 | 21.4% |
| D.不同意 | 183 | 10.45% |
| E.非常不同意 | 102 | 5.82% |
| 本题有效填写人次 | 1752 |  |

41. 我认为我所使用的设备很稳定、安全。（I recognize that the device using for the online EFL class is stable and secure.）	[单选题]

| 选项 | 小计 | 比例 |
| --- | --- | --- |
| A.非常同意 | 486 | 27.74% |
| B.同意 | 590 | 33.68% |
| C.不一定 | 385 | 21.97% |
| D.不同意 | 177 | 10.1% |
| E.非常不同意 | 114 | 6.51% |
| 本题有效填写人次 | 1752 |  |

42. 我认为我所使用的设备运行很快，不卡顿。（I recognize that the device using for the online EFL class runs smoothly and fast.）	[单选题]

| 选项 | 小计 | 比例 |
| --- | --- | --- |
| A.非常同意 | 565 | 32.25% |
| B.同意 | 600 | 34.25% |
| C.不一定 | 325 | 18.55% |
| D.不同意 | 163 | 9.3% |
| E.非常不同意 | 99 | 5.65% |
| 本题有效填写人次 | 1752 |  |

43.我认为我的课程是结构设计合理。（I recognize that the online EFL course structure is reasonable.) [单选题]

| 选项 | 小计 | 比例 |
| --- | --- | --- |
| A.非常同意 | 619 | 35.33% |
| B.同意 | 584 | 33.33% |
| C.不一定 | 284 | 16.21% |
| D.不同意 | 172 | 9.82% |
| E.非常不同意 | 93 | 5.31% |
| 本题有效填写人次 | 1752 |  |

44.我的课程得到有效的呈现。（（I have effectively presented the online EFL course for my students.) [单选题]

| 选项 | 小计 | 比例 |
| --- | --- | --- |
| A.非常同意 | 562 | 32.08% |
| B.同意 | 594 | 33.9% |
| C.不一定 | 311 | 17.75% |
| D.不同意 | 188 | 10.73% |
| E.非常不同意 | 97 | 5.54% |
| 本题有效填写人次 | 1752 |  |

45.我的授课内容做到与时俱进，实时更新。（I recognize that the online EFL course is up-to-date.) [单选题]

| 选项 | 小计 | 比例 |
| --- | --- | --- |
| A.非常同意 | 553 | 31.56% |
| B.同意 | 608 | 34.7% |
| C.不一定 | 304 | 17.35% |
| D.不同意 | 193 | 11.02% |
| E.非常不同意 | 94 | 5.37% |
| 本题有效填写人次 | 1752 |  |

46.我对学生的学习问题能做到回复及时。（ I can give prompt responses to students.) [单选题]

| 选项 | 小计 | 比例 |
| --- | --- | --- |
| A.非常同意 | 610 | 34.82% |
| B.同意 | 590 | 33.68% |
| C.不一定 | 291 | 16.61% |
| D.不同意 | 174 | 9.93% |
| E.非常不同意 | 87 | 4.97% |
| 本题有效填写人次 | 1752 |  |

47.我对课堂的整体表现表示满意。（I’m satisfied with the online EFL class.) [单选题]

| 选项 | 小计 | 比例 |
| --- | --- | --- |
| A.非常同意 | 579 | 33.05% |
| B.同意 | 619 | 35.33% |
| C.不一定 | 291 | 16.61% |
| D.不同意 | 172 | 9.82% |
| E.非常不同意 | 91 | 5.19% |
| 本题有效填写人次 | 1752 |  |

48.我很享受在线英语学习课堂。（I feel enjoyable in the online EFL classroom.) [单选题]

| 选项 | 小计 | 比例 |
| --- | --- | --- |
| A.非常同意 | 549 | 31.34% |
| B.同意 | 575 | 32.82% |
| C.不一定 | 334 | 19.06% |
| D.不同意 | 203 | 11.59% |
| E.非常不同意 | 91 | 5.19% |
| 本题有效填写人次 | 1752 |  |

49.我会将我的在线英语课堂授课方式推荐分享给其他人。（I will recommend the way to having an online EFL class for others.) [单选题]

| 选项 | 小计 | 比例 |
| --- | --- | --- |
| A.非常同意 | 479 | 27.34% |
| B.同意 | 594 | 33.9% |
| C.不一定 | 358 | 20.43% |
| D.不同意 | 206 | 11.76% |
| E.非常不同意 | 115 | 6.56% |
| 本题有效填写人次 | 1752 |  |

50.我认为在线英语学习提高了学生英语水平。（I think that students in the online EFL classroom has been improved.) [单选题]

| 选项 | 小计 | 比例 |
| --- | --- | --- |
| A.非常同意 | 451 | 25.74% |
| B.同意 | 578 | 32.99% |
| C.不一定 | 387 | 22.09% |
| D.不同意 | 210 | 11.99% |
| E.非常不同意 | 126 | 7.19% |
| 本题有效填写人次 | 1752 |  |

51.我认为在线英语课堂为我节约了很多学习时间。（I think the online EFL class saves me a lot of time.) [单选题]

| 选项 | 小计 | 比例 |
| --- | --- | --- |
| A.非常同意 | 526 | 30.02% |
| B.同意 | 600 | 34.25% |
| C.不一定 | 344 | 19.63% |
| D.不同意 | 182 | 10.39% |
| E.非常不同意 | 100 | 5.71% |
| 本题有效填写人次 | 1752 |  |

52.我认为在线英语课堂将我和同学们紧密联系起来了。（I think the online EFL class connects me with my students.) [单选题]

| 选项 | 小计 | 比例 |
| --- | --- | --- |
| A.非常同意 | 373 | 21.29% |
| B.同意 | 404 | 23.06% |
| C.不一定 | 377 | 21.52% |
| D.不同意 | 380 | 21.69% |
| E.非常不同意 | 218 | 12.44% |
| 本题有效填写人次 | 1752 |  |

53.我认为我关注在线英语教学整体水平与质量。（I concern for the overall teaching quality of the online EFL class.) [单选题]

| 选项 | 小计 | 比例 |
| --- | --- | --- |
| A.非常同意 | 485 | 27.68% |
| B.同意 | 619 | 35.33% |
| C.不一定 | 349 | 19.92% |
| D.不同意 | 196 | 11.19% |
| E.非常不同意 | 103 | 5.88% |
| 本题有效填写人次 | 1752 |  |

54.我认为在线英语课堂让英语这门课程获得成功。（I think that the online EFL class help s gain success in English teaching.) [单选题]

| 选项 | 小计 | 比例 |
| --- | --- | --- |
| A.非常同意 | 449 | 25.63% |
| B.同意 | 591 | 33.73% |
| C.不一定 | 360 | 20.55% |
| D.不同意 | 238 | 13.58% |
| E.非常不同意 | 114 | 6.51% |
| 本题有效填写人次 | 1752 |  |

55.对于在线英语课程结束后遇到的问题，我可以独立处理。（As for the problems encountered after the EFL class, I can deal with them without any prompt help-desk service.) [单选题]

| 选项 | 小计 | 比例 |
| --- | --- | --- |
| A.非常同意 | 400 | 22.83% |
| B.同意 | 591 | 33.73% |
| C.不一定 | 378 | 21.58% |
| D.不同意 | 242 | 13.81% |
| E.非常不同意 | 141 | 8.05% |
| 本题有效填写人次 | 1752 |  |

56.您认为如何可以让学生更好的融入在线英语课堂？（How will the students be engaged in the online EFL class?) [填空题]

填空题数据请通过下载详细数据获取

57.请选出6个您觉得对学生在线英语学习最有价值的学习因素（多选题）:(Please choose 6 of the following items as the most valuable online EFL learning elements.) [多选题]

| 选项 | 小计 | 比例 |
| --- | --- | --- |
| A. Understanding the course materials | 525 | 29.97% |
| B. Fluent and free internet connect | 754 | 43.04% |
| C. Being self-discipline and properly managing time | 704 | 40.18% |
| D. A silent place for studying | 453 | 25.86% |
| E. Teacher presence | 744 | 42.47% |
| F. Understanding the professor | 529 | 30.19% |
| G. Participating in student-student communications | 647 | 36.93% |
| H. Teacher-student interaction | 873 | 49.83% |
| I. Reviewing course content | 477 | 27.23% |
| J. Completing assignments | 508 | 29% |
| K. Obtaining feedback from professor | 663 | 37.84% |
| L. Obtaining feedback from assessed work | 1074 | 61.3% |
| M. Taking practice examinations | 1059 | 60.45% |
| N. Practicing problem solving ability | 797 | 45.49% |
| O. Others | 32 | 1.83% |
| 本题有效填写人次 | 1752 |  |

58.请您列举您当前面临的在线英语授课的困难。（Please list some impediments of the online EFL teaching.) [填空题]

填空题数据请通过下载详细数据获取

59.请您列举提升学生在线英语学习效果的建议(角度:硬件设备与技术、人力资源、教学质量、教学内容、教学相关服务、网络、课程设计、教师教学技巧等）:（Please list some recommendations on promoting the online EFL class for students，infrastructure and technology, human resources, quality, content and services, network enablers, curriculum design, teacher skills etc.) [填空题]

填空题数据请通过下载详细数据获取

60.请补充您认为该研究需要拓展的角度:（Please list some recommendations on this research in case of not covering those paramount perspectives.) [填空题]

填空题数据请通过下载详细数据获取
